# Satisfaction of staff of Swiss insurance companies with medical appraisals: a cross sectional study

**DOI:** 10.1186/1756-0500-4-83

**Published:** 2011-03-28

**Authors:** Klaus Eichler, Daniel Imhof, Yvonne Bollag, Susanna Stöhr, Niklaus Gyr, Holger Auerbach

**Affiliations:** 1 Winterthur Institute of Health Economics, Zurich University of Applied Sciences, Winterthur, Switzerland; 2 Academy of Swiss Insurance Medicine (ASIM), University Hospital Basel, Switzerland

## Abstract

**Background:**

A high quality of timely delivered medical appraisals is crucial for social and other insurances to judge possible occupational reintegration measures for patients with medical conditions who are in danger to lose their job. However, little is known about the satisfaction of staff of insurance companies with medical appraisals that they have commissioned.

Our questionnaire survey prospectively included all medical appraisals arriving at Swiss insurances from FEB to APR 2008. We assessed the satisfaction of the commissioner with medical appraisals performed by medical assessors. In addition, we evaluated the contribution of several factors to overall satisfaction. The unit of sample was the medical appraisal.

**Findings:**

We analysed 3165 medical appraisals, 2444 (77%) of them from the public disability insurance, 678 (22%) from private accident, liability and loss of income insurances and 43 (1%) from other insurances. Overall satisfaction of staff of insurance companies in Switzerland was high, but satisfaction of the disability insurance with appraisals was generally lower compared to satisfaction of private insurances. The staff of the disability insurance judged time for preparation as too long in 30%. For staff of private insurance companies 20% of appraisals were not "worth its price". Well-grounded and comprehensible conclusions were the single most important factor for high overall satisfaction (OR 10.1; 95%-CI: 1.1-89.3).

**Conclusions:**

From the viewpoint of staff of insurance companies, a relevant part of medical appraisals arrives too late. Medical assessors have to take the specific needs of insurances into account, to perform more appraisals with sound conclusions in due time.

## Background

Social insurances, such as the disability insurance or loss of income insurances, play an important role in Switzerland for patients with chronic medical conditions, who are in danger to lose their job. By help of medical appraisals, insurances try to get a complete picture of deficiencies and resources of such patients to judge occupational reintegration measures, for example in case of musculoskeletal or psychiatric disorders [1]. Accident and liability insurances, too, rely on medical appraisals to judge the patient situation in case of damages. Well indicated and timely reintegration measures, based on the conclusions of appraisals, can help to maintain the occupational status or avoid chronification and invalidity.

A high quality of timely delivered medical appraisals is crucial for staff of insurances to base their decisions on possible reintegration measures on solid ground [2]. Social and other insurances commission external physicians from different medical specialties as assessors to perform such medical appraisals. Assessors synthesize information from medical and psycho-social history as well as from patient examination, conclude on the overall situation of the patient and reply to key questions of insurances.

However, little is known about the satisfaction of staff of Swiss insurance companies with medical appraisals that they have commissioned. This is surprising, as more than 50 Mio Euros are spent every year solely for the generation of medical appraisals for the Swiss disability insurance [3]. Only a small pilot study of the Swiss insurance against occupational accidents and diseases (Suva) has been done, which showed frequent deficiencies of the assessed appraisals [4].

Thus, we assessed the satisfaction of staff of Swiss insurance companies with medical appraisals over a broad range of insurance areas. In addition, we evaluated the contribution of several factors to overall satisfaction of the commissioner.

## Methods

We conducted a cross-sectional questionnaire survey. The survey was part of a larger comprehensive study ("Medizinische Gutachtensituation in der Schweiz", MGS-study) [5]. In this article, we report about the satisfaction of staff of Swiss insurance companies with medical appraisals in more detail.

### Included insurances and medical appraisals

We prospectively included all consecutive medical appraisals with arrival at Swiss insurances from February 1st to April 30th, 2008. The date of commission of the appraisals was not relevant. Included Swiss insurance companies comprised the Swiss disability insurance (a public and compulsory insurance responsible for occupational reintegration measures and invalidity pensions), loss of income insurances (responsible for payments due to intermediate inability to work), accident insurances (responsible for benefits due to damages) and liability insurances (responsible for third party liabilities).

A medical appraisal was defined as any written medical assessment (with or without direct patient contact) used for appraisal of patients with insurance claims due to acute or chronic medical conditions. Thus, second opinion appraisals of previous medical certificates and appraisals for court cases were also included. We excluded reports about bio-mechanical issues in road accidents, interim reports of treatment progress for sickness insurances or simple physician attestations.

### Questionnaire development and data collection

The questionnaire was developed in a stepwise approach. Initially, we implemented an expert board of specialists for medical appraisals with practice knowledge from different fields (medical expertise; design of teaching programs for medical assessors, disability and sickness insurance knowledge; forensic expertise; experience as patient ombudsman). A literature search revealed no suitable validated questionnaire that was applicable in our context. To assure relevance of the items of the questionnaire, the expert group initially defined domains, where problems in the medical appraisal field typically occur (such as coordination between medical expert and insurance staff; formal requirements; response to key questions; usefulness of the appraisal for the commissioner). For each domain, several draft items were formulated. Draft items were amended in the expert group in an iterative process and additional useful items from the literature were incorporated. To deem face and content validity of the items and comprehensibility of formulations by persons outside the expert group, staff of six insurance companies from different fields (such as officers, insurance physicians) checked the items and amendments were made accordingly. Reliability of the questionnaire was not formally tested, but items were grouped along to the usual work-flow of appraisal generation to allow standardised response for insurance staff.

Finally, a pilot test was performed by several participating insurance companies to test the electronic version of the questionnaire using authentic appraisals. The scope was on data entry problems and possible technical difficulties, as well as on comprehensibility of items. No relevant new input for item content or formulations was generated.

The final questionnaire comprised 41 questions, including 10 questions that explored the satisfaction of the commissioner with the final appraisal. Responding was possible on a 3 to 4 point likert scale. The remaining items of the questionnaire covered insurance characteristics, process information and patient data. The questionnaire was translated into the two other main Swiss languages, French and Italian, by translators with bi-lingual mother tongue and medical background. A second bi-lingual translator checked for content consistence.

Insurance staff answered the questionnaires anonymously in a prospective manner when the companies received the appraisals. As this was a country wide study, several measures were implemented to increase data quality and participation rate (such as teaching sessions and continuous data controlling). Ethical approval was provided by the local ethics committee.

### Statistical analysis

For our descriptive analysis, we used means (SD) and medians (IQR with 25%-75% percentiles) for continuous variables and proportions for categorical data. For inferential analysis we applied parametric and non-parametric tests. The unit of analysis was the medical appraisal. For comparison of predefined subgroups we grouped appraisals of the Swiss disability insurance as "disability insurance" and appraisals of private accident, liability and loss of income insurances as "private insurances". These groups often represent different patient problems (chronic morbidity vs. damage events) as well as different organisational and legal features (public, compulsory insurance vs. private insurance companies). The two groups together comprised 98.6% of all included appraisals. Appraisals from the psychiatric specialty comprised another predefined subgroup, as our expert group judged psychiatric appraisals as specifically complex. For this subgroup, we included only mono-disciplinary psychiatric appraisals to avoid contamination from other specialties. To evaluate the contribution of single factors to overall satisfaction, we performed a multivariable logistic regression analysis (dependent variable: "very satisfied with appraisal" yes/no; examples of independent variables: "good formal structure and content" yes/no, "conclusions sound and comprehensible" yes/no; with controlling for potential confounders such as patient age, sex and involved number of medical specialties per appraisal). Data analysis was done with SPSS for Windows, version 18.0 (SPSS Inc., Chicago, Illinois); for regression analysis we used Stata 9.0 (StataCorp 2004, Stata Statistical Software, College Station, TX).

## Results

### Medical appraisals and patients

We included 3165 medical appraisals for analysis. Of those, 2444 appraisals (77%) had been commissioned by the disability insurance and 678 appraisals (22%) by private insurances (43 appraisals of other insurances; Table 1). Data quality was high with mean missing data of 1% (range for single items: 0% to 6.9%). 65% of appraisals were mono-disciplinary (i.e. only one medical specialty involved), 14% were bi-disciplinary and 20% were poly-disciplinary (three up to seven specialties). Staff of insurances, which collected the data, was most often insurance physicians (42%) or officers with experience in processing of applications and appraisals (32%). Many of them (52%) had a specific job experience of between 6 and more than 20 years. Mean time interval from commissioning to arrival of appraisals at the insurance company was 20.8 (SD 16.1) weeks for the disability insurance, compared to 11.6 (SD 16.8) weeks for private insurances (p < 0.01).

**Table 1:** Characteristics of Medical Appraisals and Patients.

Type of appraisal	N = 3165*
Appraisal for disability insurance, No. (%)	2444 (77)
Appraisal for private insurances (private accident insurances; liability insurances; loss of income insurances), No. (%)	678 (22)
Other appraisals (non-private loss of income, accident or occupational disease insurances; or no data entry), No. (%)	43 (1)

**Number of involved medical specialties per appraisal**	

1 specialty (mono-disciplinary appraisal), No. (%)	2072 (65)
2 specialties (bi-disciplinary appraisal), No. (%)	437 (14)
3 up to 7 specialties (poly-disciplinary appraisal), No. (%)	656 (21)

**Time interval needed for generation of appraisal**	

Appraisal for disability insurance, weeks (SD)	20.8 (16.1)
Appraisal for private insurances, weeks (SD)	11.6 (16.8)

**Patients assessed with appraisal**	

Mean age (SD)	45.9 (10.5)
Women, No. (%)	1606 (51)

**Employment status **(prior to the problem that lead to medical appraisal)	

Self-employed, No. (%)	206 (7)
Employed (non-specialist function), No. (%)	1590 (50)
Employed (specialist function), No. (%)	927 (29)
Employed (superior function), No. (%)	100 (3)
Other (work at home; looking for job; education), No. (%)	342 (11)

* The unit of analysis is the medical appraisal. Due to missing data, subgroups comprise less than all cases per group.

The mean patient age was 45.9 years and 68% of the patients (2115/3165) were in the age group between 41 and 60 years. 50.7% of the patients were female. In the past, most of the patients had been employed (n = 2617; 83%) or self-employed (n = 206; 6.5%).

### Satisfaction with medical appraisals

Overall satisfaction of staff of insurance companies with appraisals was high (34% [1073/3165] "very satisfied" and 54% [1704/1365] "satisfied"; 9.2% [291/3165] "not satisfied"; for 3% of appraisals staff answered "no judgement possible"; Table 2). Single domains showed a mixed picture of satisfaction. Similarly high satisfaction rates as for overall satisfaction were expressed for a variety of items (such as for "inclusion of pre-existing information and former appraisals" or "response to key questions"). Staff of insurance companies was less satisfied with timeliness of preparation and the relationship between usefulness of results for decision making and price of the appraisal. While for 71% the time interval for preparation was judged as adequately, for every fourth appraisal (26%) time for preparation was judged as too long. Only for three quarters of the cases (76%) the appraisal was deemed as "worth its price", for 14% this was not or only partly the case in the personal judgement of the insurance staff.

**Table 2:** Satisfaction of Staff of Insurance Companies with Medical Appraisals

	Total	Appraisals for disability insurance*	Appraisals for private insurances*	Psychiatric appraisals (mono-disc.)
	N = 3165	n = 2444	n = 678	n = 1159
**Item 1: Inclusion of pre-existing information**				

Sufficiently addressed, No. (%)	2816 (89.0)	2198 (89.9)	584 (86.1)	1012 (87.3)
Not sufficiently addressed, No. (%)	121 (3.8)	88 (3.6)	25 (3.7)	46 (4.0)
No judgement possible, No. (%)	228 (7.2)	158 (6.5)	69 (10.2)	101 (8.7)

**Item 2: Formal structure**				

Well structured, No. (%)	2986 (94.3)	2299 (94.1)	651 (96.0)	1070 (92.3)
Badly structured, No. (%)	111 (3.5)	84 (3.4)	21 (3.1)	57 (4.9)
No judgement possible, No. (%)	68 (2.1)	61 (2.5)	6 (0.9)	32 (2.8)

**Item 3: Presentation of content**				

Comprehensibly presented, No. (%)	2893 (91.4)	2210 (90.4)**^#^**	646 (95.3)**^#^**	1050 (90.6)
Not comprehensibly presented, No. (%)	186 (5.9)	156 (6.4)	25 (3.7)	73 (6.3)
No judgement possible, No. (%)	86 (2.7)	78 (3.2)	7 (1.0)	36 (3.1)

**Item 4: Response to key questions**				

Response complete, No. (%)	2667 (84.3)	1985 (81.2)**^#^**	647 (95.4)**^#^**	971 (83.8)
Response not complete, No. (%)	387 (12.2)	357 (14.6)	23 (3.4)	145 (12.5)
No judgement possible, No. (%)	111 (3.5)	102 (4.2)	8 (1.2)	43 (3.7)

**Item 5: Conclusions**				

Well founded and comprehensible, No. (%)	2774 (87.6)	2115 (86.5)**^#^**	633 (93.4)**^#^**	1000 (86.3)
Badly founded and not sufficiently comprehensible, No. (%)	278 (8.8)	228 (9.3)	33 (4.9)	107 (9.2)
No judgement possible, No. (%)	113 (3.6)	101 (4.1)	12 (1.8)	52 (4.5)

**Item 6: Additional queries to the assessor**				

No additional queries necessary, No. (%)	2721 (86.0)	2075 (84.9)**^#^**	615 (90.7)**^#^**	987 (82.2)
Additional queries necessary, No. (%)	241 (7.6)	196 (8.0)	37 (5.5)	85 (7.3)
No judgement possible, No. (%)	203 (6.4)	173 (7.1)	26 (3.8)	87 (7.5)

**Item 7: Volume of the appraisal**				

Too short, No. (%)	137 (4.3)	98 (4.0)	32 (4.7)	57 (4.9)
Just as short/as long as necessary, No. (%)	2705 (85.5)	2053 (84.0)**^#^**	624 (92.0)**^#^**	979 (84.5.0)
Too long, No. (%)	197 (6.2)	171 (7.0)	19 (2.8)	68 (5.9)
No judgement possible, No. (%)	126 (4.0)	122 (5.0)	3 (0.4)	55 (4.7)

**Item 8: Time interval for preparation of appraisal**				

Adequately long, No. (%)	2255 (71.2)	1637 (67.0)**^#^**	583 (86.0)**^#^**	908 (78.3)
Too long, No. (%)	819 (25.9)	737 (30.2)	77 (11.4)	228 (19.7)
No judgement possible, No. (%)	91 (2.9)	70 (2.9)	18 (2.7)	23 (2.0)

**Item 9: Is the appraisal „worth its price"?^$^**				

Yes, No. (%)	1539 (76.4)	1137 (76.0)	375 (78.1)	521 (82.6)
Partly, No. (%)	236 (11.7)	144 (9.6)	87 (18.1)	70 (11.1)
No, No. (%)	55 (2.7)	43 (2.9)	7 (1.5)	16 (2.5)
No judgement possible, No. (%)	185 (9.2)	173 (11.6)	11 (2.3)	24 (3.8)

**Item 10: Overall satisfaction**				

Very satisfied, No. (%)	1073 (33.9)	788 (32.2)**^#^**	272 (40.1)**^#^**	396 (34.2)
Satisfied, No. (%)	1704 (53.8)	1316 (53.8)	373 (55.0)	601 (51.9)
Not satisfied, No. (%)	291 (9.2)	244 (10.0)	32 (4.7)	117 (10.1)
No judgement possible, No. (%)	97 (3.1)	96 (3.9)	1 (0.1)	45 (3.9)

The unit of analysis is the medical appraisal: *Disability insurance (DI) and private insurances (PI) comprise 98.6% of all analysed medical appraisals; **^#^**Significant difference between DI and PI (p < 0.05; chi-square test); **^$^**Only appraisals included, for which insurance staff was aware of the price.

Satisfaction of the staff of the Swiss disability insurance with appraisals of medical assessors for their patients was generally lower compared to private insurances. The most striking differences were found for the domains "timeliness of appraisals" (not satisfied: disability insurance 30.2% vs. private insurances 11.4%; p < 0.001), "completeness of response to key questions" (incomplete response: 14.6% vs. 3.4%; p < 0.001) and "soundness and comprehensibility of conclusions" (not sufficient: 9.3% vs. 4.9%; p < 0.001). Overall satisfaction with 1159 mono-disciplinary psychiatric appraisals was basically the same as for the total population (86% vs.87% "very satisfied" or "satisfied"). Also for items that covered specific sub-domains, no relevant differences were found in comparison to the total population.

The single most important factor for high overall satisfaction in our multivariable regression analysis was "sound and comprehensible conclusions" of the appraisal (odds ratio for high overall satisfaction: OR 10.1, 95%-CI: 1.1 to 89.3), followed by "timeliness of preparation" (OR 5.9, 95%-CI: 4.3 to 8.0) and a balanced relationship between price and usefulness of the appraisal ("worth its price": OR 5.7, 95%-CI: 3.2 to 9.9; Table 3). Factors such as formal structure or inclusion of pre-existing information had no significant influence on overall satisfaction in our sample.

**Table 3:** Adjusted Odds Ratios for high Overall Satisfaction with Medical Appraisals

	Odds ratio (95%-CI)*	p-value
**Item 1: Inclusion of pre-existing information**		

Not sufficiently addressed	Reference	
Sufficiently addressed	1.27 (0.51-3.16)	0.61

**Item 2: Formal structure**		

Badly structured	Reference	
Well structured	1.48 (0.27-8.16)	0.66

**Item 3: Presentation of content**		

Not comprehensibly presented	Reference	
Comprehensibly presented	1.60 (0.17-14.8)	0.68

**Item 4: Response to key questions**		

Response not complete	Reference	
Response complete	2.80 (1.62-4.84)	<0.001

**Item 5: Conclusions**		

Badly founded and not sufficiently comprehensible	Reference	
Well founded and comprehensible	10.1 (1.14-89.3)	0.04

**Item 6: Additional queries to the assessor**		

Additional queries necessary	Reference	
No additional queries necessary	3.68 (1.50-9.07)	0.005

**Item 7: Volume of the appraisal**		

Too short or too long	Reference	
Just as short/as long as necessary	2.16 (1.26-3.71)	0.005

**Item 8: Time interval for preparation of appraisal**		

Too long	Reference	
Adequately long	5.85 (4.26-8.02)	<0.001

**Item 9: Is the appraisal „worth its price"?**		

No or partly	Reference	
Yes	5.66 (3.24-9.88)	<0.001

**Number of disciplines involved in appraisal**		

Mono-disciplinary appraisal	Reference	
Bi- or Poly-disciplinary appraisal	1.36 (1.17-1.58)	<0.001

**Personal characteristics of insurance staff**		

Years of professional experience (per additional year)	0.99 (0.98-1.01)	0.41

*Other covariates included in the multivariable analysis: patient age, patient sex. The odds-ratio (95%-Confidence-interval, CI) indicates the chance that staff of insurance companies is "very satisfied" overall (Item 10). The unit of analysis is the medical appraisal.

## Discussion

This is the first country-wide Swiss study that shows empirical data about the satisfaction of staff of public and private insurance companies with medical appraisals for diverse insurance areas. Thus, this health services research data can provide deeper insight into real world conditions of insurance medicine, which is relevant for decision makers. Overall, staff of public and private insurance companies in Switzerland are satisfied with medical appraisals, but time demand for preparation was judged as too long for every fourth appraisal. Well-grounded and comprehensible conclusions were the single most important factor for high overall satisfaction.

Satisfaction with appraisals for the disability insurance was generally lower compared to appraisals for private insurances. This finding correlates well with the time interval needed for generation of appraisals as measured in our study (mean time interval for disability insurance: 20.8 weeks; for private insurances: 11.6 weeks). Even though appraisals covering questions of disability are often very complex, nearly half a year of mean waiting time for appraisals was judged as too long (dissatisfaction rates: 30% vs. 11%). This has a significant influence on overall satisfaction, as our data have shown.

Interestingly, satisfaction with mono-disciplinary psychiatric appraisals was not relevantly different from satisfaction with other appraisals. This may underline, that even in patients with possibly complex psychiatric problems commissioners are equally satisfied with appraisals, if conclusions are comprehensible and the appraisal task is finished in due time.

Comparison with published data about the satisfaction of staff of Swiss insurances with medical appraisals is limited, as only highly selected appraisals [6] or only legal aspects have been judged in the past [7]. Our study has confirmed some of the findings of the Swiss pilot study about 102 mostly mono-disciplinary appraisals of patients with occupational accidents [4]. Satisfaction had been similarly high for some items (for example, 89% of appraisals with complete response to key questions), but long waiting times for every third appraisal had been criticized, as well. Studies from Scandinavia have also highlighted time consuming waiting times and partly insufficient content of certificates for the social insurance [8] or coordination problems between social insurance officers and assessors [9]. With our quantitative approach, we could confirm central findings of this valuable qualitative data for the Swiss setting.

Our study has some limitations. Firstly, we do not have data to judge the completeness of the number of appraisals for each of the participating insurance companies during the study period. However, considerable efforts have been made to involve key players of the insurances to install an effective internal controlling. Secondly, the coverage of all medical appraisals in the study period was not possible. Some private Swiss insurance companies have not participated in the study or contributed only some few appraisals for organisational reasons. However, we were able to analyse a broad range of appraisals from different insurances areas from all three language regions of Switzerland. We believe that the data are sufficiently representative to display the current situation in Switzerland concerning medical appraisals. Thirdly, the major part of the included appraisals came from the disability insurance. While this reflects the big volume of appraisals for the public, compulsory disability insurance in Switzerland, overall results have to be interpreted cautiously. The results for private insurances are partly different, as shown by the data. Finally, insurance staff may be biased towards higher satisfaction rates, as they were not blinded to the medical professionals who they had invited for appraisal writing. Due to resource limitations, however, multiple checks by additional blinded insurance staff were not possible. Furthermore, blinding was not intended, to ensure data entry by the specific officer/physician, who was familiar with details of the patient case to reliably judge key items, such as coordination issues.

### Implications for practice and research

The results of our study should be incorporated into the continuous education programme of physicians, who perform medical appraisals. This can increase their awareness that sound and comprehensible conclusions in the medical appraisal are of outstanding importance. Specifically in court cases this is of relevance, as non-medical legal specialist have to base their decisions on comprehensible medical conclusions [7]. Furthermore, standards of time limits for generation of appraisals should be established. Any time loss due to late appraisals will reduce the probability of early reintegration measures for the patient, if indicated.

A specific sub-project of the MGS-study has assessed the correlation between overall satisfaction of the commissioners and the professional quality of appraisals as judged by trained medical experts [10]. Commissioners were able to identify good appraisals sufficiently well, but had problems to identify low quality appraisals. Thus, the high overall satisfaction of insurance staff with appraisals, as seen in our data, may give an optimistic picture concerning the quality of medical appraisals. Satisfaction research in hospitals has shown, that overall satisfaction rates can be partly misleading [11]. Given these findings, continuous educational measures are also necessary for the staff of insurances. This will enable them to select assessors who provide state of the art appraisals in due time. Nevertheless, assessment of satisfaction from the viewpoint of insurance staff remains important. Some relevant issues can only be judged by the "clients" (i.e. the staff of insurances), such as coordination issues [9] and timeliness.

A follow-up study would contribute valuable information about improvements over time. For example, in the context of current reforms of the Swiss disability law, that promotes reintegration measures to avoid invalidity pensions [12]. Similar studies in the neighbour countries of Switzerland may create useful information to learn from each other. We hypothesize, that conditions in countries such as Germany or Austria may not be long apart.

## Conclusions

Medical assessors have to take the specific needs of insurances concerning medical appraisals into account. Sound conclusions as well as timeliness of preparation are essential to facilitate well indicated reintegration measures in due time.

## Competing interests

The authors declare that they have no competing interests.

## Authors' contributions

KE participated in the design of the study and its coordination, collected data, performed the statistical analysis and drafted the manuscript. DI participated in the design of the study and the statistical analysis. YB, SS, NG and HA participated in the design of the study and its coordination and collected data. All authors revised the draft manuscript critically for important content and read and approved the final version.

## References

[B1] Anonymous Leitlinien der Schweizerischen Gesellschaft für Versicherungspsychiatrie für die Begutachtung psychischer Störungen Schweiz Ärztezeitung 2004 85 20 1048 1051

[B2] Ludwig C Anforderungen an Gutachten - Anforderungen an Gutachter Schweiz Ärztezeitung 2006 87 23 1035 1036

[B3] Häuptli L Bund zahlt Millionen für IV-Gutachten Neue Zürcher Zeitung. Zürich 2008 16

[B4] Ludwig C Gutachtenqualität im Unfallversicherungsbereich Suva - Med Mitteilungen 2006 77 5 16

[B5] Auerbach H Bollag Y Eichler K Imhof D Stöhr S Gyr N Medizinische Gutachten in der Schweiz im Jahr 2008: Eine Querschnittstudie zur Marktsituation und Qualitätssicherung Suva Medical 2010 9 21

[B6] Meine J Die ärztliche Unfallbegutachtung in der Schweiz - Erfüllt sie die heutigen Qualitätsanforderungen? Swiss Surg 1998 4 53 57 9587228

[B7] Blindenbacher E Was erwartet der Jurist vom medizinischen Gutachter? Arzt und Praxis 1997 2 28 33

[B8] Ydreborg B Ekberg K Nilsson K Swedish social insurance officers' experiences of difficulties in assessing applications for disability pensions--an interview study BMC Public Health 2007 7 128 10.1186/1471-2458-7-128 17597536 PMC1913505

[B9] Thorstensson CA Mathiasson J Arvidsson B Heide A Petersson IF Cooperation between gatekeepers in sickness insurance - the perspective of social insurance officers. A qualitative study BMC Health Serv Res 2008 8 231 10.1186/1472-6963-8-231 18992160 PMC2586030

[B10] Stohr S Bollag Y Auerbach H Eichler K Imhof D Fabbro T Gyr N Quality assessment of a randomly selected sample of Swiss medical expertises - a pilot study Swiss Med Wkly 2011 141 w13173 21374530 10.4414/smw.2011.13173

[B11] Gerteis M Edgman-Levitan S Daley J Delbanco T Through the patient's eyes 1993 San Francisco, CA: Jossey-Bass Inc

[B12] Bundesamt für Sozialversicherungen Argumentarium: JA zur Eingliederungsversicherung IV 2007 Eidgenössisches Dept. des Inneren. Bern

